# Supplementary material for: Sketch, capture and layout phylogenies
Source: PLoS Comput Biol. 2025 Dec 15;21(12):e1013805. doi: 10.1371/journal.pcbi.1013805 (PMC12714275; doi:10.1371/journal.pcbi.1013805)

Overall, this manuscript is extremely well written and introduces a novel layout algorithm implemented in an accessible desktop application. PhyloSketch allows users to visualise phylogenetic trees and networks with support for both combining and transfer views. The novel layout algorithm uses simulated annealing to get around the computational complexisties of the network layout problem. Additionally, PhyloSketch includes methods to extract topologies from images when the tree files are not shared with a publication.

I have a few minor comments related to the manuscript itself which I have listed in the following table. All other comments are related to the PhyloSketch app.

**Line number**	**Comment**
58	All leaves should have in-degree = 1
133	Move definition of LCA to first occurrence e.g. line 95
136	Is it possible for the user to control the parameters of the SA algorithm e.g. initial and goal temperatures?
136	Are the layouts produced by SA reproducible? I see in the benchmark there is a large amount of variation in the replicates.
163	Write "…we assume that every tree edge or transfer-acceptor edge" for clarity
Figure 3	In IcyTree this is an "ancestral recombination graph" not "time tree".
203	I would like to see some more disscusiton of the utility of these methods. Are you able to provide real world uses of the capture methods? Can you, for example, examine capture trees from papers with known topologies and show that PhyloSketch accurately reconstructs the underlying newick?
Fig ure 4	What's the contribution of reticulations? Please generate another plot keeping the number of tips constant and vary the proportion of reticulations.
Figure 4	Please provide the technical specifications of the laptop.
211	How accurate are the branch lengths from the captured tree? Is possible to use the scale bar to calibrate branch lengths?

244	I would like to see some more concrete evidence as to why your new approach improves on Dendroscope. How does it compare computationally? How often can source and target of transfer edges be placed on the same level? E.g. does this only happen a fraction of the time or in every tree?
257	Please include a screenshot of the app with the major features annotated.
257	Why “should” it work on mobile? Is this something that has been tested? Do you have any details about mobile use?
259	I don’t think there is any point in mentioning AI without any insights. I recommend removing this line and ending with a stronger conclusion.
Acknowledgments	Banu Cetinkaya (@banucetinkaya) has made several commits to the codebase on Github but is not acknowledged.

App related comments

I tested the PhyloSketch App on macOS and found the installation process to be simple. I was able to capture a small tree from an image and use the layout algorithms effectively. However, I noticed several issues related to the UI and larger trees/networks that inhibited my analysis.

- As a new user I find the discoverability of features in the app to be low. Adding the names of menus next to the icons would help. An in-app tutorial/walkthrough would also be helpful otherwise it seems the app is not useable without repeatedly referring to the manual.
- The capture algorithm seems brittle; it works well on some images but fails almost completely on others. I think some image preprocessing tools could really help the capture process e.g. letting the users change the image contrast in the app. Additionally, it would be helpful to expose the parameter of the capture algorithm to let users dynamically adapt the capture process to different images.
- In manuscripts trees are often presented in panels combined with other text or figures. These complex figures can make tree capture difficult. It would be useful to have more options to crop the captured image in PhyloSketch (current crop is only square to the view window size) or add a method to define an area of interest in which the capture

algorithm is applied.

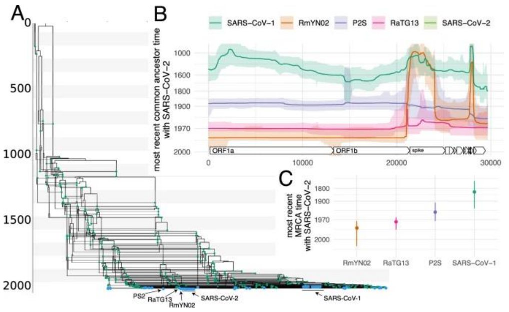

- While the app works well on small to medium sized trees, I found it difficult to capture large topologies (>250 samples), requiring a significant amount of manual work to complete the capture. I would appreciate some examples or tutorials on capturing large trees or guidelines for the upper limits. The size of phylogenies reported in papers is increasing and so it is likely that PhyloSketch will be used on larger trees in the future.
- I see an offset error where the captured phylogeny is to the top-left of the images. Not sure if this is a feature or a bug? But it makes it a little difficult to make additional accurate modifications without first repositioning the entire tree.

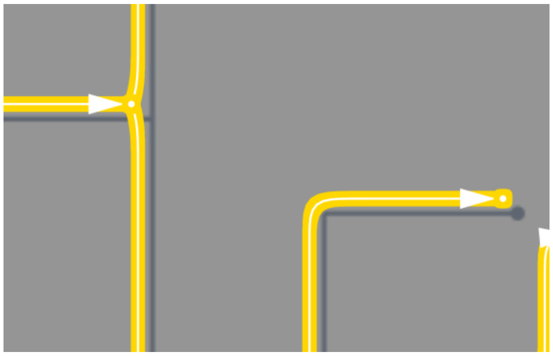

- The app becomes sluggish when selecting many nodes or edges (>200) maybe there should be a warning about the limitations of larger trees about be a warning for large trees?
- It would be useful to have a vertical spacing control for the rectangular layout e.g. if there are a lot of labels they overlap and cannot be read.
- While I can resize the nodes in the Node Style section, I cannot change the shape.
- Tip labels are not rotated in the circular layout

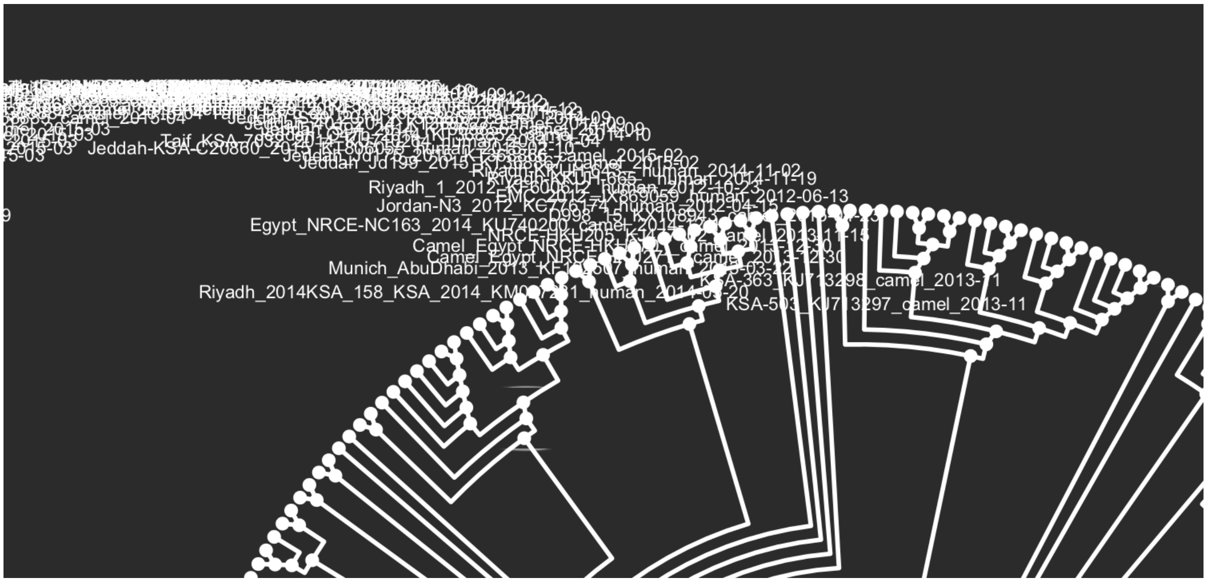

Supplement: Attachment — Submitted filename: Paper Huson.pdf [file pcbi.1013805.s001.pdf]
