# Supplementary material for: Sketch, capture and layout phylogenies
Source: PLoS Comput Biol. 2025 Dec 15;21(12):e1013805. doi: 10.1371/journal.pcbi.1013805 (PMC12714275; doi:10.1371/journal.pcbi.1013805)
Supplement: Attachment — Submitted filename: review PhyloSketch.pdf [file pcbi.1013805.s002.pdf]

Review of "Sketch, capture and layout phylogenies"

Reviewer

October 8, 2025

Overall Assessment

This manuscript presents a valuable contribution to phylogenetic network visualisation. The writing is clear and effectively makes complex algorithmic concepts accessible to the broader research community. PhyloSketch fills a significant gap in the field by offering the first interactive tool that combines three key functionalities: sketching phylogenetic networks from scratch, capturing networks from published images, and computing optimised layouts for complex reticulate structures.

The software is user-friendly, easily installable, and compatible across platforms, including mobile devices, enhancing its potential for widespread adoption. The open-source nature and comprehensive implementation make it accessible to researchers. The authors have undertaken considerable effort to create this interactive tool, which addresses an unresolved problem in the field.

Major Comments

- The paper would greatly benefit from the inclusion of quantitative evaluation metrics for the image capture workflow.
- A more thorough benchmarking against existing phylogenetic network visualisation tools is needed, particularly regarding running times and memory usage, beyond the brief qualitative mentions.
- Please provide details on simulated annealing parameters, sensitivity analysis, and guidance on parameter selection and result quality assessment. Specifically, indicate which parameters users need to fix to ensure satisfactory algorithm performance.

Minor Comments

- The user experience could be improved by including example datasets in the GitHub repository and manual.
- Scalability issues with a larger number of taxa could be mentioned in the discussion.

Conclusion

This paper makes a significant and timely contribution to computational phylogenetics by providing a comprehensive, user-friendly tool for phylogenetic network visualisation. The technical approach is sound, the implementation is robust, and the potential impact on the research community is substantial. With the suggested improvements, particularly in evaluation and user guidance, this work could establish PhyloSketch as the standard tool for interactive phylogenetic network analysis. The authors' vision for incorporating AI-based approaches in future versions positions this work at the cutting edge of computational biology tool development, demonstrating a clear path for continued innovation in this critical area.
